# Evaluating the effectiveness and safety of Xiyanping injection for severe pneumonia: a target trial emulation protocol using real-world data

**DOI:** 10.3389/fphar.2026.1862861

**Published:** 2026-08-06

**Authors:** Meng Qiao, Chaoren Tan, Wenxi Peng, Chao Lei, Qiang Zhang, Chengjun Ban, Yanming Xie, Zhifei Wang

**Affiliations:** 1 Institute of Basic Research in Clinical Medicine, China Academy of Chinese Medical Sciences, Beijing, China; 2 Institute of Acupuncture and Moxibustion, China Academy of Chinese Medical Sciences, Beijing, China; 3 Guang’anmen Hospital, China Academy of Chinese Medical Sciences, Beijing, China; 4 The First Affiliated Hospital of Henan University of Chinese Medicine, Zhengzhou, China; 5 Dongzhimen Hospital, Beijing University of Chinese Medicine, Beijing, China

**Keywords:** causal inference, dose-response relationship, real-world evidence, severe pneumonia, target trial emulation, Xiyanping injection

## Abstract

**Background:**

Severe pneumonia remains a major public health challenge characterized by high mortality. Xiyanping Injection (XYP) is a Traditional Chinese Medicine injectable preparation whose core active constituent is andrographolide sulfonate, a sulfonated derivative of andrographolide extracted from *Andrographis paniculata* (Burm.f.) Wall. ex Nees [Acanthaceae; *Andrographis paniculatae herba*]. XYP exhibits anti-inflammatory, antiviral, antibacterial, antipyretic, and immunomodulatory activities. Although preliminary studies indicated the potential efficacy of XYP as an adjunctive therapy, its real-world effectiveness, safety, and optimal dosing in severe pneumonia remain uncertain. To bridge the gap between theoretical efficacy and real-world clinical benefit, and to overcome the methodological biases inherent in traditional observational studies, target trial emulation (TTE) is needed to generate high-quality real-world evidence.

**Methods:**

We will emulate a pragmatic target trial using multicenter electronic health records. Time zero will be aligned with the date of confirmed severe pneumonia diagnosis, and a new-user design will be implemented to reduce immortal time bias and prevalent user bias. We will compare XYP combined with standard care versus standard care alone. The primary outcomes are 28-day all-cause mortality and in-hospital mortality. Missing data will be handled using machine-learning-based imputation tailored to variable types. The clone-censor-weight method will serve as the primary analytical strategy. The instrumental variable approach will be used as a secondary method to explore residual confounding. We will further apply causal forests to estimate conditional average treatment effects and to explore potential treatment-effect heterogeneity and high-benefit subgroups. A multistage dose-response analysis will examine the association between continuous XYP dosing and clinical outcomes to explore potential dosing patterns and therapeutic windows.

**Discussion:**

This protocol applies a rigorous causal inference framework to evaluate the real-world effectiveness and safety of XYP as adjunctive treatment for severe pneumonia. The study is expected to provide real-world evidence to inform future evaluation of XYP in real-world clinical practice and to provide a replicable TTE-based methodological template for real-world evaluation of other Traditional Chinese Medicine injections.

**Clinical Trial Registration:**

https://itmctr.ccebtcm.org.cn/, Identifier ITMCTR2026000820.

## 1 Introduction

Severe pneumonia is a critical global public health challenge with high morbidity and mortality (Xie et al., 2023). The Global Burden of Disease Study estimates that lower respiratory tract infections (primarily pneumonia) cause approximately 250 million incident cases and more than 2.6 million deaths each year worldwide (Wang et al., 2024; Niu et al., 2025). Approximately 10%–15% of community-acquired pneumonia (CAP) cases progress to severe pneumonia, with reported mortality of 30%–50% (Jenkins-Lonidier, 2021). In China, severe CAP constitutes 13.58%–20.05% of hospitalized CAP cases, posing a particularly significant threat to older adults (Komiya et al., 2024; Chinese Medical Doctor Association, Division of Emergency Physicians et al., 2025).

Current management of severe pneumonia relies on early empirical antimicrobial therapy, together with individualized respiratory support and selected adjunctive treatments (Martin-Loeches et al., 2023). However, several challenges limit outcomes in routine practice. First, rising antimicrobial resistance among key pathogens (e.g., *Streptococcus pneumoniae* and *Mycoplasma pneumoniae*), including resistance to macrolides, compromises the effectiveness of standard regimens (Sundaresh et al., 2021). Second, severe pneumonia is frequently accompanied by immune dysregulation (e.g., hyperinflammation and lymphocyte depletion), yet safe and effective targeted immunomodulatory options remain limited (Sundaresh et al., 2021). Third, the role of adjunctive corticosteroids remains debated because of uncertainty regarding patient selection and concerns about secondary infections (Ceccato et al., 2021). These limitations highlight the need to evaluate additional adjunctive therapeutic strategies, particularly for patients facing multifactorial barriers to recovery.

Xiyanping injection (XYP) is a Traditional Chinese Medicine (TCM) injection widely used for respiratory infectious diseases in China (Zhou and Zhang, 2024). The active pharmaceutical ingredient of XYP is andrographolide sulfonate, a water-soluble derivative produced by sulfonation of andrographolide, which is extracted from the medicinal plant *Andrographis paniculata* (Burm.f.) Wall. ex Nees [Acanthaceae; *Andrographis paniculatae herba*]. This structural modification improves its aqueous solubility and may increase systemic exposure (Li Z. et al., 2025). Unlike conventional crude herbal extracts, XYP is a standardized formulation composed of andrographolide sulfonates and water for injection, without added excipients. XYP was approved by the National Medical Products Administration of China in 2002 (Approval No. Z20026249). Pharmacological studies suggest that XYP has antiviral, antibacterial, anti-inflammatory, and immunomodulatory activities (Cai et al., 2020; Liu et al., 2023; Jiang et al., 2023; Jiang and Ma, 2024; Rao et al., 2024). Several small-sample randomized controlled trials (RCTs) have indicated that XYP, when administered as an adjunctive therapy, can ameliorate clinical symptoms and abbreviate disease duration in patients with severe pneumonia, preliminarily demonstrating its clinical efficacy in older adults and pediatric patients with severe pneumonia, as well as those complicated by respiratory failure (Zhu and Lu, 2018; Liu and Li, 2019; Zeng et al., 2022; Zhou and Zhang, 2024).

Despite these preliminary findings, substantial uncertainty remains regarding the clinical role of XYP in severe pneumonia. First, the current evidence is dominated by small-sample RCTs, and their restrictive eligibility criteria and controlled settings may limit external validity in the heterogeneous populations encountered in critical care. Second, while prior studies have suggested potential efficacy, the drug’s effectiveness and practical value in non-interventional, real-world settings require validation. In addition, real-world dosing varies considerably (typical daily doses reported as 50–500 mg/day), and the dose-response relationship remains unclear. The potential heterogeneity of treatment effects, i.e., which patient subgroups are most likely to benefit, also remains insufficiently characterized (Li et al., 2023). Addressing these practical questions is essential to inform standardized use and to define where XYP may fit within comprehensive severe pneumonia management.

The safety profile of XYP also warrants careful evaluation in real-world practice. Available evidence suggests that the overall incidence of XYP-associated adverse drug reactions is relatively low, with reported events mainly involving allergic or systemic reactions, including cutaneous manifestations, gastrointestinal symptoms, and mild respiratory or cardiovascular reactions (Li Z. et al., 2025). However, XYP has clinically relevant risks of drug–drug interactions and physicochemical incompatibilities. For example, admixture with certain drugs (e.g., acyclovir or ganciclovir) may reduce the content of the main active ingredient(s), whereas mixing with others (e.g., ambroxol hydrochloride injection) may increase insoluble particulates, potentially triggering pyrogenic or anaphylactoid reactions (Li Z. et al., 2025). In addition, XYP may inhibit CYP3A4 activity and thereby affect the metabolism of drugs such as lopinavir/ritonavir; it was also reported to alter azithromycin metabolism, potentially resulting in pharmacokinetic interactions (Li Z. et al., 2025). Therefore, co-administration of XYP with other injectable agents in the same infusion container is contraindicated, and compatibility guidance should be followed in clinical practice (Li Z. et al., 2025).

RCTs are widely regarded as the gold standard for estimating causal effects, because randomization can balance measured and unmeasured confounders in expectation (Matthews et al., 2022; Lambourg, 2024). However, strict eligibility criteria and highly controlled implementation may limit their ability to represent the complexity of routine care (Matthews et al., 2022; Lambourg, 2024). To address this limitation in external validity, observational studies using real-world data (RWD) have become an important source of evidence. Increasingly, real-world studies (RWS) and RCTs are viewed as complementary rather than competing approaches (Morales and Arlett, 2023). RWS can address questions that are difficult to study in trials and can also inform the design of subsequent confirmatory RCTs by providing preliminary estimates of effect size and feasibility (Liu et al., 2024). However, conventional observational designs are vulnerable to bias, which can undermine causal interpretation (Matthews et al., 2022; Scola et al., 2023). To strengthen causal inference from RWD, the target trial emulation (TTE) framework was proposed (Hernán and Robins, 2016; Gomes et al., 2022). The central idea of TTE is to explicitly specify and emulate the key design elements of a hypothetical pragmatic trial, thereby reducing selection bias, confounding, and information bias that commonly affect observational analyses (Gomes et al., 2022; Andrade, 2025). Grounded in the counterfactual framework and causal graphical models, TTE aims to estimate effects that more closely approximate the causal effects that would be obtained under the target trial (Yiu et al., 2022).

Accordingly, this pre-specified protocol will apply the TTE framework to multicenter electronic health records (EHRs) from the National Health Big Data Center. Before data extraction and analysis, we detail the methodological framework designed to systematically evaluate the real-world effectiveness and safety of XYP combined with standard care versus standard care alone in patients with severe pneumonia. Specifically, we will estimate the causal effects of XYP on 28-day all-cause mortality and in-hospital mortality (primary outcomes), and will assess secondary outcomes including clinical recovery milestones, dynamic biomarker changes, and pre-defined safety events relevant to real-world use. This study is intended to address practical questions regarding clinical use, while providing quantitative inputs for the design of future confirmatory RCTs. Ultimately, the findings may inform future optimization of comprehensive treatment strategies for severe pneumonia and contribute methodological evidence for the evidence-based evaluation of TCM.

## 2 Methods

### 2.1 Study design and data source

This study protocol delineates a retrospective observational cohort study based on EHRs, conducted within the TTE framework. The objective is to evaluate the real-world effectiveness and safety of XYP combined with standard care versus standard care alone in adult patients with severe pneumonia.

The study will leverage large-scale, real-world EHR data sourced from Jiangsu Province, China. This database covers more than 30,000 healthcare institutions across all 13 prefecture-level cities in the province, capturing the clinical trajectories of an underlying population of approximately 80 million residents. The anticipated study inclusion period spans from 1 January 2020, to 31 December 2024.

**FIGURE 1:**
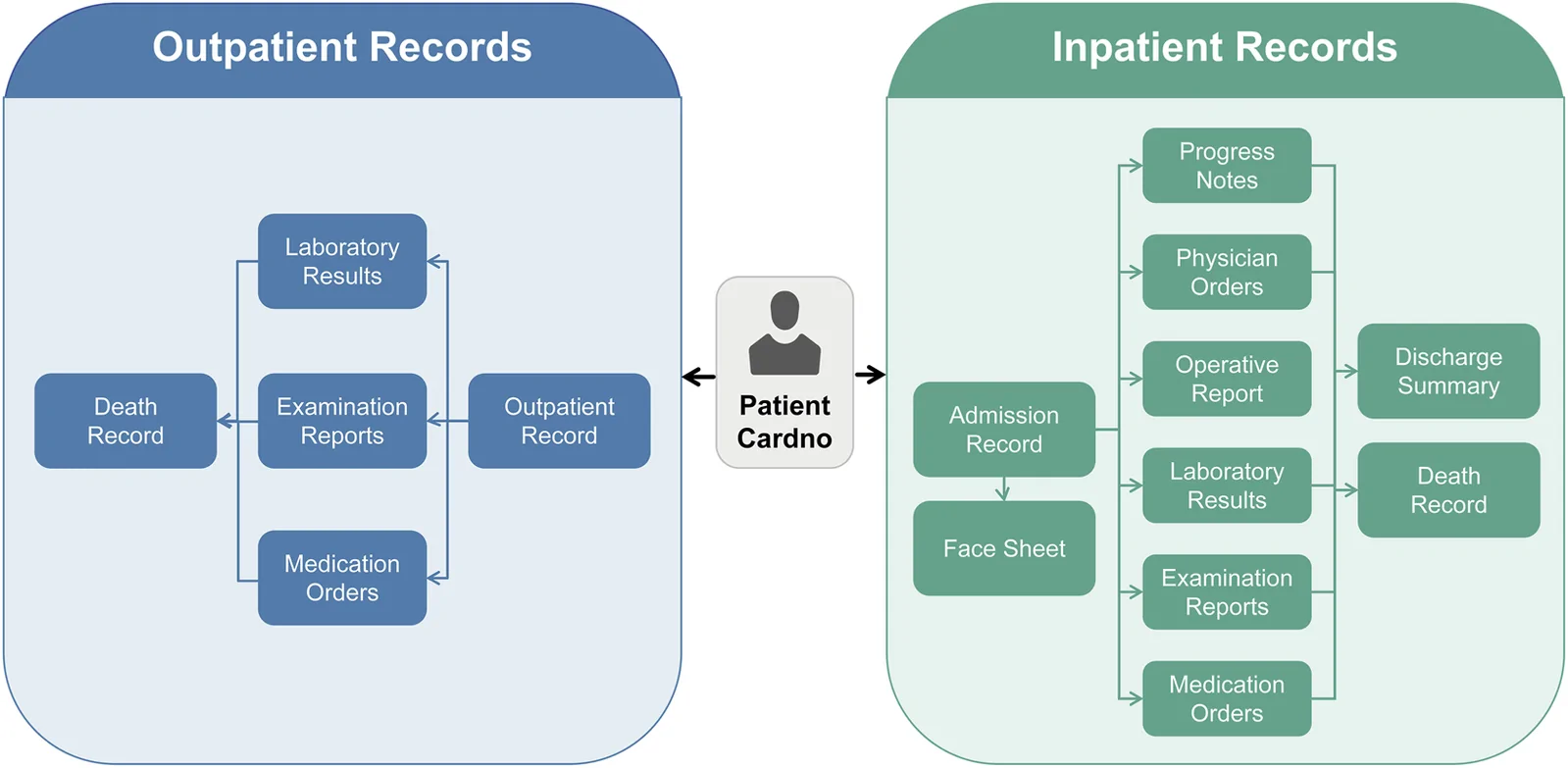
Schematic representation of the EHR data architecture and linkage strategy.

To construct longitudinal patient trajectories, records from distinct clinical encounters will be linked using a unique, encrypted patient identifier (cardno). These linked longitudinal records will enable the retrospective ascertainment of pre-diagnosis medication histories, the precise timing of post-diagnosis treatment initiation, outcome ascertainment from linked hospitalization and survival records, and the repeated capture of post-baseline clinical measurements required for the primary analytical approach. In strict adherence to ethical regulations and data privacy protocols, all individual-level patient information will be de-identified prior to extraction and analysis. The structured data primarily include demographic information, diagnostic records, treatment histories (including medication administration and clinical interventions), laboratory test results, imaging reports, and survival data (Figure 1).

### 2.2 Target trial emulation framework

The study applies the TTE framework to explicitly define the epidemiological design components. The protocol of the hypothetical pragmatic RCT and the corresponding emulation strategy utilizing observational EHR data are summarized in Table 1.

**TABLE 1:** Core components of the target trial and its emulation strategy.

Protocol component	Target trial protocol	Target trial emulation strategy
Eligibility criteria	Adult (≥18 years) patients diagnosed with severe pneumonia Exclusions: Non-infectious pulmonary diseases, severe baseline comorbidities New users: no XYP within 3 months prior to enrollment	Adult (≥18 years) patients diagnosed with severe pneumonia Exclusions: Non-infectious pulmonary diseases, severe baseline comorbidities, documented XYP use within 3 months prior to the confirmed diagnosis, documented allergy or hypersensitivity to XYP, andrographolide, or Andrographis paniculata, and incomplete core data
Treatment strategies	Intervention strategy group: XYP + standard care Control strategy group: Standard care alone	Intervention strategy group: Initiate XYP combined with standard care within a 48-h grace period. Control strategy group: Standard care alone (no XYP). Ascertained via detailed inpatient medication administration logs and electronic prescription records
Assignment procedures	Patients are randomly assigned to either intervention strategy group or control strategy group at baseline	Patients are observationally “assigned” based on actual physician prescription
Time zero and follow-up	Time zero: Date of randomization and severe pneumonia diagnosis. Follow-up ends at death, discharge, or 28 days	Time zero: Date of confirmed severe pneumonia diagnosis. A 48-h grace period is applied for treatment initiation. Follow-up is identical to the target trial
Outcome	Primary: 28-day all-cause mortality, in-hospital mortality Secondary: Clinical recovery milestones, dynamic biomarker shifts, and safety events	Identical to the target trial
Causal contrast	Intention-to-treat effect or per-protocol (PP) effect	Observational analogue of the per-protocol effect of initiating XYP within the 48-h grace period versus standard care alone

### 2.3 Eligibility criteria

#### 2.3.1 Inclusion criteria

Patients will be included if they meet all of the following criteria:

1. Confirmed diagnosis of severe pneumonia: Defined according to the Infectious Diseases Society of America/American Thoracic Society consensus guidelines, requiring the presence of at least one major criterion or three or more minor criteria (Mandell et al., 2007):
  Major criteria: (a) Requirement for invasive mechanical ventilation; (b) Septic shock requiring vasopressors despite adequate fluid resuscitation.
  Minor criteria: (a) Respiratory rate ≥30 breaths/min; (b) Arterial oxygen pressure/fraction of inspired oxygen ratio ≤250 mmHg; (c) Multilobar infiltrates; (d) Confusion and/or disorientation; (e) Blood urea nitrogen ≥20 mg/dL; (f) Leukopenia (white blood cell count <4.0 ×10^9^/L); (g) Thrombocytopenia (platelet count <100 × 10^9^/L); (h) Hypothermia (core temperature <36 °C); (i) Hypotension requiring aggressive fluid resuscitation.
2. Age: ≥18 years.
3. Data completeness: Availability of complete core clinical trajectory data, including baseline demographics and detailed treatment information for the current visit.

#### 2.3.2 Exclusion criteria

Patients meeting any of the following criteria will be excluded:

1. Non-infectious or specific pulmonary diseases: Including pulmonary fibrosis, active pulmonary tuberculosis, interstitial pneumonia, and pulmonary tumors.
2. Severe comorbidities: Presence of conditions likely to substantially alter baseline mortality risk, including severe arrhythmias, decompensated cirrhosis, nephrotic syndrome, primary immunodeficiency diseases, and acquired immunodeficiency syndrome.
3. Competing interventions: Documented administration of XYP within 90 days prior to diagnosis, or concurrent use of other TCM injections with similar pharmacological properties (e.g., Tanreqing injection, Reduning injection) that may confound the effectiveness assessment.
4. History of allergy: Documented history of allergy or hypersensitivity to *A. paniculata* (Burm.f.) Wall. ex Nees [Acanthaceae; *A. paniculatae herba*] andrographolide, or XYP.
5. Missing Data: Severe missingness of critical survival data, precluding the robust evaluation of the primary outcomes.

### 2.4 Treatment strategies and time zero

#### 2.4.1 Intervention and control strategies

The study will adopt a new-user design to mitigate prevalent user bias and ensure the correct temporal ordering of covariate assessment and treatment initiation (Zhao and Wang, 2022; Yasrebi-de Kom et al., 2025). Specifically, eligible patients must have no documented prescription of XYP during the 90 days preceding the confirmed diagnosis, verified through retrospective query of the linked longitudinal medication records.

The intervention strategy is defined as the administration of XYP combined with standard care (including antibiotics, antivirals, respiratory support, and corticosteroids if indicated), while the control strategy consists of standard care alone. Time zero (baseline) (Hernán et al., 2022) is defined as the date of the confirmed diagnosis of severe pneumonia. To ensure causal temporality and reduce immortal time bias, this date serves as the common starting point of follow-up for all patients.

#### 2.4.2 Botanical drug information and quality assurance

To improve transparency and reproducibility of intervention reporting, the botanical reporting in this protocol follows the Consensus-based reporting guidelines for the Phytochemical Characterisation of Medicinal Plant extracts (Heinrich et al., 2022) and the Four Pillars of Best Practice in Ethnopharmacology (Heinrich et al., 2020). XYP (Approval No. Z20026249) is an approved, commercially available pharmaceutical product; therefore, the research team did not handle the raw botanical materials. The botanical authentication of the source plant, *A. paniculata* (Burm.f.) Wall. ex Nees [Acanthaceae; *A. paniculatae herba*], and the deposition of voucher specimens were performed by the sole manufacturer (Jiangxi Qingfeng Pharmaceutical Co., Ltd., China) in compliance with the Good Manufacturing Practice mandated by the National Medical Products Administration. As a widely cultivated crop, its procurement fully complies with the principles of the Nagoya Protocol and Convention on International Trade in Endangered Species of Wild Fauna and Flora regulations.

Regarding production, XYP is manufactured by extracting andrographolide from the aerial parts of the plant, followed by sulfonation to yield sodium andrographolide sulfonate as the main active component in an aqueous injectable formulation. Manufacturing and quality control adhere to the National Quality Standard [WS-10863 (ZD-0863)-2002-2011Z]. Under this standard, the content of sodium andrographolide sulfonate should be 90.0%–110.0% of the labeled amount (calculated on a dried basis). Accordingly, XYP exposure in this study corresponds to a marketed product with standardized quality specifications, supporting batch-to-batch comparability for pharmacoepidemiologic evaluation.

#### 2.4.3 Grace period

Within the TTE framework, the grace period for initiating XYP in the intervention strategy is set to 48 h after diagnosis. The interval from time zero to the first XYP administration order is ascertained using the linked longitudinal medication records. Because severe pneumonia can progress rapidly and parenteral therapy is typically initiated early, this window is intended to reflect routine clinical decision-making while allowing time for initial diagnostic work-up (e.g., microbiological testing). With the primary outcome being 28-day all-cause mortality, a relatively short grace period ensures a robust causal linkage between the intervention and the acute outcome. This specification also aims to balance clinical plausibility with the need to retain an adequate sample size for analysis.

### 2.5 Outcomes

#### 2.5.1 Primary effectiveness outcomes

The primary effectiveness outcomes are 28-day all-cause mortality (death from any cause within 28 days after time zero) and in-hospital mortality (all-cause death occurring during the index hospitalization).

#### 2.5.2 Secondary effectiveness outcomes

Secondary effectiveness outcomes include clinical recovery trajectories and biomarker changes. Clinical milestones include length of hospital stay (days from time zero to discharge or death), intensive care unit (ICU) length of stay (days in the ICU among ICU-admitted patients), and duration of mechanical ventilation (total hours or days of invasive or non-invasive ventilatory support, as recorded in the EHR). Key biomarkers will be extracted at pre-specified time points (day 14 and day 28 after time zero, where available), including white blood cell count, C-reactive protein, procalcitonin, arterial partial pressure of oxygen, arterial pH, arterial partial pressure of carbon dioxide, and serum albumin.

#### 2.5.3 Safety outcomes

Safety outcomes include newly identified organ dysfunction and severe clinical complications after time zero. Organ function will be assessed using hepatic markers (alanine aminotransferase (ALT), aspartate aminotransferase (AST), and total bilirubin (TBIL)), renal markers (serum creatinine (SCr) and blood urea nitrogen (BUN)), and cardiac markers (cardiac troponin I (cTnI) and N-terminal pro-B-type natriuretic peptide (NT-proBNP)). In addition, severe clinical events will include respiratory failure, acute respiratory distress syndrome, septic shock, multiple organ dysfunction syndrome, and pulmonary embolism. Suspected allergic reactions and severe anaphylaxis will be monitored as pre-specified safety events given the parenteral route of administration (Tong et al., 2022).

To further address cumulative exposure and interaction-related safety concerns, the cumulative exposure level of XYP during the index hospitalization will be summarized based on the total administered dose, treatment duration, and dosing frequency. Concomitant Western medicines commonly used in the management of severe pneumonia, including antibiotics, antivirals, corticosteroids, vasoactive agents, and respiratory-support-related medications, will be extracted from medication administration records. Particular attention will be paid to concomitant drugs with documented metabolic or physicochemical compatibility concerns. Newly developed or worsening hepatic and renal dysfunction after time zero will be evaluated using both baseline and post-baseline ALT, AST, TBIL, SCr, and BUN values.

### 2.6 Covariates

To control for baseline confounding, covariates will be extracted for each patient and measured at or prior to time zero to avoid adjustment for post-baseline variables. Pre-specified covariates include: (1) demographics and socioeconomic factors (age, sex, marital status, ethnicity, occupation, educational attainment, admission department, payment method, and lifestyle factors such as smoking and alcohol use); (2) clinical characteristics, including admission vital signs (temperature, blood pressure, heart rate, respiratory rate, and consciousness), disease severity (CURB-65), microbiological etiology (bacterial, viral, fungal, or mixed, where available), baseline organ dysfunction (ALT, AST, TBIL, SCr, BUN, cTnI and NT-proBNP), and comorbidities (e.g., hypertension, diabetes, chronic obstructive pulmonary disease, coronary heart disease, cerebral infarction, and anemia); and (3) baseline care processes, including antimicrobial classes (e.g., β-lactams, macrolides, fluoroquinolones), systemic corticosteroids, vasoactive agents, and initial respiratory support (non-invasive or invasive mechanical ventilation), and institution-level adjunctive treatment patterns, as recorded before or at time zero.

### 2.7 Data quality control

#### 2.7.1 Logical verification

Rule-based data checks will be conducted to improve data quality. Temporal consistency will be assessed to ensure that treatment initiation occurs after diagnosis and that outcome events are recorded after time zero. Plausibility checks will verify whether continuous variables (e.g., age, length of stay, laboratory values) fall within clinically reasonable ranges. Consistency among related variables will be evaluated (e.g., ICU length of stay not exceeding total hospital stay). Records violating these rules will be flagged and, where feasible, resolved through audit of the original EHR entries; otherwise, they will be excluded from the relevant analyses.

#### 2.7.2 Missing data mechanism and imputation

We will first summarize the extent and patterns of missingness for baseline covariates, laboratory variables, treatment information, and primary outcome ascertainment. Missingness proportions will be reported overall and by treatment strategy, and, where relevant, according to key observed factors such as hospital, calendar year, and baseline disease severity. Patients with severe missingness of critical survival data will be excluded according to the eligibility criteria; primary mortality outcomes will not be imputed in the primary analysis.

The primary analysis will assume that missing baseline covariates and laboratory variables are missing at random (MAR), conditional on observed clinical and care-process information, including patient characteristics, baseline disease severity, hospital-level factors, calendar time, treatment status, and auxiliary variables predictive of missingness (Little and Rubin, 2019). This assumption is considered more plausible than missing completely at random (MCAR) in real-world EHR data, because missingness of laboratory tests and clinical measurements is often related to observed care processes, disease severity, and hospital practice patterns. However, missing not at random (MNAR) mechanisms may exist if missingness depends on unobserved disease severity, undocumented clinical judgment, or unmeasured patient characteristics. Violations of the MAR assumption could bias treatment-effect estimates through residual confounding, differential covariate measurement, or selection of patients with more complete clinical records.

Missing baseline covariates and laboratory variables will be handled primarily using machine learning–based imputation, with the specific algorithm selected according to variable type and observed missingness patterns. Tree-based methods, such as random forest or gradient-boosted decision trees, will be used where appropriate to accommodate non-linear associations and interactions (Sun et al., 2023; Benhamza et al., 2025; Mushava and Murray, 2022). The imputation model will include the treatment indicator, all covariates used for confounding adjustment, variables used in the censoring and weighting models, hospital-level characteristics, calendar time, and auxiliary variables predictive of missingness. Diagnostic checks, including range checks and distributional comparisons between observed and imputed values, will be performed to assess the clinical plausibility of imputed data.

### 2.8 Sample size estimation

Sample size estimation accounts for superiority testing on the primary outcome, potential effective sample size loss due to clone–censor–weight (CCW) procedures, and model stability for weighted survival analyses. Unless otherwise specified, sample sizes in this section refer to unique individuals before cloning.

First, the baseline sample size was calculated based on 28-day all-cause mortality. Based on prior literature, the 28-day all-cause mortality rate under standard care is approximately 20% (Martin-Loeches et al., 2023). Assuming that XYP is associated with a 5% absolute reduction (to 15%), with a two-sided α of 0.05, 80% power, and 1:1 allocation, the required sample size for comparing two independent proportions is 1,806 participants (903 per group).

Second, under the CCW approach, artificial censoring due to protocol deviations can reduce the effective sample size, and inverse probability of censoring weights (IPCW) may inflate variance. To account for this, we applied an *a priori* inflation factor of 1.5, reflecting a conservative adjustment commonly used when weight variability is anticipated. After inflation, the required cohort size increases to approximately 2,710 individuals.

Third, for model stability, the primary analysis will use weighted Cox regression. Following an events-per-variable rule of thumb of ≥20 events per parameter (Althubaiti, 2023), and assuming 27 baseline covariates, at least 540 deaths are required. Assuming an average 28-day all-cause mortality of 17.5% across the cohort, this corresponds to a minimum analytical sample size of 3,086 individuals. Because this exceeds the requirement from hypothesis testing, 3,086 is adopted as the target minimum sample size.

In conclusion, this study aims to include at least 3,086 eligible adults with severe pneumonia. Given the province-wide coverage, the covered population is approximately 80 million. We anticipate that the number of eligible cases during 2020–2024 will be sufficient to meet this target.

### 2.9 Statistical analysis

**FIGURE 2:**
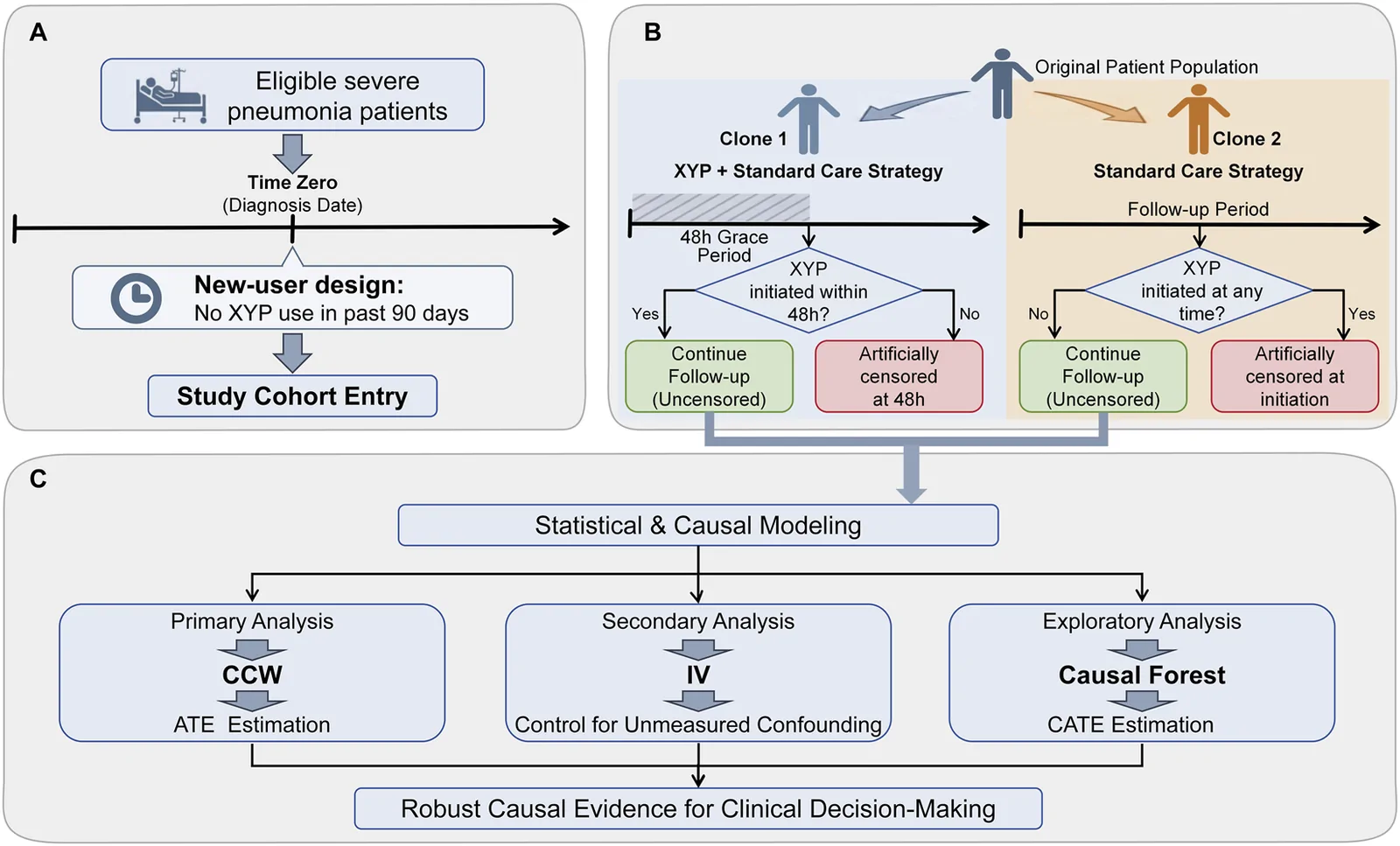
Study design and causal inference framework for target trial emulation. **(A)** Cohort entry, with the confirmed diagnosis of severe pneumonia defined as time zero and a 90-day new-user washout period. **(B)** Cloning into the XYP plus standard care and standard care-alone strategies, followed by artificial censoring according to XYP initiation during the 48-h grace period. **(C)** Primary clone-censor-weight analysis for the average treatment effect, secondary instrumental-variable analysis for unmeasured confounding, and exploratory causal-forest analysis for conditional average treatment effects. XYP, Xiyanping injection; IV, instrumental variable; CCW, clone-censor-weight; ATE, average treatment effect; CATE, conditional average treatment effect.

All analyses will be conducted using R (version 4.2.2). Key packages include survival, rms, survey, and grf. All tests will be two-sided, and P < 0.05 will be considered statistically significant. The overall analysis workflow is presented in Figure 2.

#### 2.9.1 Causal estimands and identification assumptions

The primary CCW analysis targets the per-protocol effect of initiating Xiyanping Injection (XYP) combined with standard care within a 48-h grace period versus standard care alone on 28-day all-cause mortality and in-hospital mortality. Identification of this effect relies on several assumptions (Cole and Hernán, 2008). First, conditional exchangeability requires that, after adjustment for measured baseline prognostic factors and treatment predictors, treatment strategy and artificial censoring are independent of the potential outcomes. This assumption is supported by the new-user design, alignment of time zero to the date of severe pneumonia diagnosis, and adjustment for a rich set of baseline and pre-treatment covariates; informative censoring will be addressed using inverse probability of censoring weights. Second, positivity requires that each treatment strategy and remaining uncensored status have non-zero probabilities within relevant covariate strata; this will be evaluated using propensity-score and weight distributions. Third, consistency requires that the observed outcome under the received strategy corresponds to the potential outcome under the specified strategy, supported by standardized intervention definitions, the 48-h grace period, exclusion of concomitant TCM injections with similar indications, and use of a single standardized marketed XYP product. Finally, correct specification of the censoring, weighting, and outcome models is assumed; its plausibility will be assessed through model diagnostics, covariate balance after weighting, weight stability, and pre-specified sensitivity analyses.

The secondary IV analysis estimates an IV-adjusted treatment effect of XYP initiation on mortality, reflecting the effect among patients whose treatment choice is influenced by institutional prescribing preference. This estimand is complementary to, but not identical with, the per-protocol estimand targeted by the primary CCW analysis. Causal interpretation requires that the instrument is associated with individual XYP initiation, is conditionally independent of unmeasured individual-level prognostic factors after adjustment for measured patient characteristics, hospital characteristics, and calendar time, and affects mortality only through XYP initiation rather than through other aspects of care (Baiocchi et al., 2014). Because the exclusion restriction cannot be empirically verified and institutional prescribing preference may be correlated with broader care patterns, IV estimates will be interpreted cautiously as a secondary robustness analysis rather than as confirmatory evidence.

#### 2.9.2 Primary analysis: Clone-censor-weight approach (CCW)

The primary analysis will use the CCW approach to emulate the target trial and estimate the per-protocol effect of initiating XYP within the 48-h grace period versus standard care alone, as defined in the causal framework above (Hernán and Robins, 2016). At time zero (date of severe pneumonia diagnosis), each eligible individual will be cloned and assigned to both strategies (initiate XYP vs. standard care alone). Clones will be artificially censored when their observed treatment deviates from the assigned strategy: intervention clones will be censored if XYP is not initiated within the 48-h grace period, and control clones will be censored upon initiation of XYP or other TCM injections with similar intended indications (as defined above).

To adjust for informative censoring, time-varying IPCW will be estimated (Olivier, 2026). Stabilized IPCW will be derived from pooled logistic regression models estimating the probability of remaining uncensored at each daily follow-up interval. The denominator model will include baseline covariates and time-varying covariates measured before each interval that may predict both artificial censoring and clinical outcomes. The numerator model will include treatment strategy, follow-up time, and a reduced set of baseline factors to improve weight stability. Time-varying covariates, including repeated laboratory values, vital signs, and changes in organ support, will be captured from the linked longitudinal records. Missing baseline covariates and laboratory variables will be imputed before weight estimation, and the imputed variables will be incorporated into both the censoring-weight models and outcome models, as specified in the missing-data strategy.

Weight diagnostics will include inspection of stabilized weight distributions, extreme values, percentiles, mean stabilized weights, and effective sample size. Extreme weights will be truncated at pre-specified percentiles to improve stability, and the influence of extreme weights will be assessed in sensitivity analyses where appropriate. Covariate balance before and after weighting will be assessed using standardized mean differences, with values below 0.1 generally considered indicative of acceptable balance (Austin and Stuart, 2015; Desai and Franklin, 2019). If substantial imbalance remains after weighting, alternative censoring-weight model specifications will be explored.

Treatment effects will be estimated using weighted Cox proportional hazards models, reporting hazard ratios (HRs) with 95% confidence intervals (CIs) for time-to-event outcomes (e.g., 28-day all-cause mortality). For continuous and binary secondary outcomes, weighted generalized linear models will be used as appropriate. Cumulative incidence curves and absolute risk differences will be estimated using weighted Kaplan-Meier methods.

#### 2.9.3 Secondary analysis: Instrumental variable approach (IV)

To operationalize the secondary IV analysis described above, institutional prescribing preference will be used as the instrument (Brookhart et al., 2006; Bidulka et al., 2024). It will be defined as the proportion of incident severe pneumonia patients who received XYP in the preceding 12 months within the same hospital or department. When calculating institutional prescribing preference, the index patient will be excluded where feasible to ensure that the instrument is not directly constructed from the patient’s own treatment status.

Instrument strength will be evaluated using first-stage diagnostics, including the first-stage F-statistic, with F > 10 considered supportive of instrument strength (Keane and Neal, 2023). A two-stage residual inclusion (2SRI) approach will be used (Zhang and Lewsey, 2024). In the first stage, a logistic regression model will estimate receipt of XYP as a function of the IV and baseline covariates, from which residuals will be obtained. In the second stage, these residuals and baseline covariates will be included in Cox models for time-to-event outcomes, or appropriate generalized linear models for non-time-to-event outcomes, to estimate IV-adjusted effects. IV findings will be compared with the primary CCW estimates to assess the robustness of the main conclusions to potential unmeasured confounding.

#### 2.9.4 Exploratory analysis: Causal forest approach

A causal forest model within the generalized random forest framework will be used to explore heterogeneous treatment effects by estimating the conditional average treatment effect (CATE) (Langenberger et al., 2023). Inputs will include baseline covariates, treatment assignment, and the primary outcomes, and IPCW will be incorporated as observation weights. Nuisance functions (propensity score and outcome regression) will be estimated using random forests to enable orthogonalization (Langenberger et al., 2023; Van Vogt et al., 2025). An honesty-based sample-splitting strategy will be employed to reduce overfitting (Jawadekar et al., 2023). Heterogeneity will be assessed using an omnibus calibration test based on the best linear predictor of out-of-bag CATE estimates (Langenberger et al., 2023; Van Vogt et al., 2025). Variable importance will be examined to identify baseline features contributing to effect heterogeneity. The population will then be stratified into subgroups based on predicted CATE distributions, and subgroup average treatment effects with 95% CIs will be estimated using augmented inverse probability weighting (Shiba and Inoue, 2024). Baseline clinical profiles will be compared across subgroups to characterize potential responder phenotypes.

In addition to the causal forest analysis, pre-specified exploratory subgroup analyses will be conducted to improve the clinical interpretability of treatment-effect heterogeneity. Where data permit and subgroup sample sizes are sufficient, treatment-effect estimates will be explored across strata defined by age, microbiological etiology, CURB-65 score, and baseline organ dysfunction, with particular attention to elderly patients aged ≥65 years and patients with baseline hepatic or renal dysfunction. The causal forest analysis and these subgroup analyses will be interpreted as exploratory assessments of treatment-effect heterogeneity rather than confirmatory evidence.

#### 2.9.5 Dose-response analysis

A multi-stage dose-response analysis will be conducted to evaluate the relationship between XYP dose and clinical outcomes. The exploratory analysis will be restricted to XYP users. A weighted Cox model with restricted cubic splines (RCS) will be fitted to model the non-linear association between daily labeled product dose (mg/day) and the log hazard of the primary outcome (Desquilbet and Mariotti, 2010). The model will adjust for baseline covariates; stabilized inverse probability weights will be used to reduce confounding among users. The adjusted hazard ratio curve will be plotted using the median dose (or another clinically common dose) as the reference. Subsequently, guided by RCS-identified inflection points and the empirical distribution of prescribed doses, exposure will be categorized into clinically interpretable strategies (e.g., standard care alone; standard care plus low-dose XYP; standard care plus high-dose XYP). Consistent with a pre-specified protocol, the final categorization scheme and thresholds will be determined based on the observed dose distribution and clinical interpretability. The CCW emulation will then be re-applied to compare these dose-defined strategies, with re-estimation of time-varying IPCW to address informative censoring due to deviations. Weighted Cox models will be used for pairwise comparisons, and a test for trend will be performed by modeling dose category as an ordinal variable. Because dose assignment in routine care is not randomized and may be influenced by disease severity, clinician preference, and patient tolerance, the dose-response findings will be interpreted as exploratory rather than confirmatory evidence for an optimal dosing regimen.

#### 2.9.6 Sensitivity analyses

To assess the robustness of the study findings, several pre-specified sensitivity analyses will be conducted. The potential influence of unmeasured confounding on the primary outcomes will be quantitatively evaluated by calculating the E-value (VanderWeele and Ding, 2017).

Meanwhile, to test the robustness of the grace period setting within the CCW framework, a sensitivity analysis regarding the parameter for treatment initiation will be conducted. In addition to the primary 48-h analysis, we will redefine and emulate target trials with grace periods of 24 h (representing an ultra-early and aggressive intervention scenario) and 72 h (accommodating extended clinical observation and diagnostic delays). By comparing the treatment effect estimates across these varying grace period definitions, we aim to evaluate the sensitivity of the causal conclusions to the time-window specification and further explore the timing-effect of XYP intervention.

For the primary CCW analysis, additional sensitivity analyses will assess the influence of the weighting procedure by evaluating alternative censoring-weight model specifications and the impact of extreme-weight handling. Across these specifications, we will compare treatment-effect estimates, weight distributions, effective sample size, and covariate balance to determine whether the conclusions are sensitive to model specification or unstable weights.

Furthermore, to verify the stability of the analytical models, the primary analyses will be replicated using alternative statistical approaches, including propensity score matching (PSM), propensity score stratification, and standard multivariable Cox or logistic regression models. The consistency of effect directions and the overlap of 95% CIs across the primary CCW approach, the IV analysis, and these alternative models will be systematically assessed to evaluate the robustness of the main overall treatment-effect estimates.

Finally, missing-data sensitivity analyses will be incorporated into both the primary CCW analysis and the secondary IV analysis. Treatment-effect estimates from the primary machine learning–based imputation strategy will be compared with estimates from complete-case analyses and multiple imputation by chained equations using variable-type–appropriate models (White et al., 2011). For key variables with substantial missingness, departures from the MAR assumption will be explored, where feasible, using conservative controlled-imputation or pattern-mixture specifications. Under each missing-data strategy, the CCW analysis will be repeated with re-estimated censoring weights and outcome models, and the IV analysis will be repeated with re-estimated first-stage and second-stage models. For MICE-based analyses, censoring weights and outcome models will be fitted within each imputed dataset, and treatment-effect estimates will be combined using standard multiple-imputation combining rules. Consistent effect directions and similar magnitudes across these analyses will support robustness, whereas materially different estimates will be interpreted as evidence that conclusions may be sensitive to missing-data assumptions.

## 3 Discussion

Severe pneumonia persists as a major global health challenge (Jenkins-Lonidier, 2021); despite substantial advancements in its clinical management, associated mortality rates remain high (Martin-Loeches et al., 2023). Although RCTs are widely regarded as the gold standard for evaluating the causal effects of interventions, their controlled experimental environments and rigid inclusion/exclusion criteria often result in highly selected patient cohorts. Consequently, they may not fully represent the complex and heterogeneous population of critically ill patients in real-world practice (Schünemann et al., 2006; Wang et al., 2023). Meanwhile, the proliferation of large-scale RWD and advances in causal inference methodology have provided an opportunity to address these evidence limitations, fostering a new research paradigm that emphasizes the synergy and complementarity of RCTs and RWS (Tian and Xie, 2010; Zou et al., 2016; Moccia et al., 2024). Against this backdrop, this protocol emulates a hypothetical target trial using the TTE framework applied to a multicenter EHR database. We will evaluate the real-world effectiveness and safety of XYP added to standard care, compared with standard care alone, among patients with severe pneumonia, with the goal of generating estimates that are closer to per-protocol effects under explicit assumptions. The findings are intended to inform the design of future confirmatory RCTs and to provide real-world evidence relevant to future evaluation of dose-response patterns and patient subgroups that may derive greater benefit.

XYP possesses a strong pharmacological rationale and potential clinical value in the treatment of severe pneumonia. The pharmacological basis of XYP for treating severe pneumonia lies in its multiple pharmacological effects—including immunomodulatory, anti-inflammatory, antiviral, and antibacterial properties (Tong et al., 2022; Ci et al., 2023; Zhang Y. C. et al., 2023). Through these mechanisms, it may help intervene in the cytokine storm, alleviate pulmonary inflammatory injury, and act synergistically to clear pathogens, thereby potentially improving the prognosis of severe pneumonia (Ci et al., 2023). The clinical manifestations of patients with severe pneumonia are often complex; patients may present with gastrointestinal dysfunction, such as slowed gastrointestinal motility and malabsorption, or require mechanical ventilation. These conditions severely limit the feasibility of oral medications and may compromise their absorption (Zhang M. et al., 2023). As a widely used TCM parenteral formulation, XYP can effectively bypass the limitations of oral administration in critically ill patients, enabling relatively rapid and controllable drug delivery. Administered via intravenous infusion or intramuscular injection, XYP is predominantly prescribed in inpatient settings, particularly in ICU or specialized respiratory wards. This inpatient administration paradigm aligns well with the robust data architecture of high-quality hospital EHR systems, in which medication orders and administration records are typically captured with fine temporal granularity. This high degree of data granularity and temporal fidelity can improve exposure measurement accuracy and reduce the likelihood of exposure misclassification, thereby establishing a key data foundation for the rigorous causal inference analyses detailed subsequently in this protocol.

The methodological rigor of this protocol is built on the TTE framework, the CCW approach, and prespecified data curation procedures. The TTE framework applies the protocol of a hypothetical, well-defined RCT to observational data (Hernán and Robins, 2016). By aligning “time zero” and adopting a new-user design, TTE helps avoid immortal time bias and prevalent user bias that can arise in retrospective analyses (Fu, 2023). Specifically, the new-user design includes individuals at the first time they meet eligibility criteria and assigns this moment as “time zero,” ensuring that follow-up begins from a common origin and reducing temporal imbalances between groups (Li X. Y. et al., 2025). It also mitigates prevalent user bias, whereby patients already receiving treatment may differ systematically from untreated patients at baseline (Li X. Y. et al., 2025). For effect estimation, we prespecify CCW as the primary approach. By cloning individuals across strategies, censoring deviations, and applying time-varying IPCW, CCW addresses time-related biases while retaining the full eligible cohort, thereby avoiding sample loss that can occur with matching-based designs (Li et al., 2020; Li et al., 2025 X. Y.; Perhanidis et al., 2024). This preservation is particularly crucial for research involving critically ill patients, where sample sizes are typically limited; retaining more samples helps maintain the real-world representativeness of the critical care cohort. Furthermore, given the high-dimensional and heterogeneous nature of real-world EHR data and the possibility of complex, non-linear missingness patterns, this protocol prespecifies a customized machine learning-based imputation strategy as the primary approach for handling missing baseline covariates and laboratory variables (Benhamza et al., 2025). Missing data are pervasive in observational research, and inappropriate handling may reduce statistical efficiency and introduce bias. In particular, complete-case analysis (listwise deletion) may lead to selection bias and loss of precision when data are not missing completely at random (Casiraghi et al., 2023). In contrast, machine learning methods such as Random Forest, XGBoost, and CatBoost can flexibly model non-linearities and high-order interactions in high-dimensional settings, which may improve imputation performance when conventional parametric models are misspecified (Benhamza et al., 2025). Accordingly, we will tailor the imputation procedure to variable type and observed missingness patterns, aiming to preserve key multivariable relationships and support downstream causal analyses. To enhance credibility, imputed values will be evaluated using clinical plausibility checks, including range and distribution diagnostics, and sensitivity analyses will examine the robustness of inferences under alternative missing-data assumptions.

To estimate the causal effect of XYP in severe pneumonia, we prespecify CCW as the primary analytical framework, complemented by IV analysis as a secondary approach. CCW is designed to address time-related biases, including immortal time bias, deviations from the assigned strategy, and time-varying confounding, under measured-confounding assumptions (Bhatia et al., 2025). However, unmeasured factors, such as nuanced clinical judgment, patient preferences, or institutional practice patterns may remain in routine care (Shi et al., 2024). IV analysis is therefore included to probe the potential sensitivity of the findings to unmeasured confounding, conditional on the plausibility of the IV assumptions (Bastardoz et al., 2023). Based on preliminary exploration of the study data, we observed variation in XYP prescribing patterns across facilities; the joint use of CCW and IV analyses is intended to support robustness evaluation by comparing complementary approaches rather than relying on a single method.

A prespecified sensitivity analysis plan will assess the robustness of the findings to unmeasured confounding, grace-period definitions, model specification, and missing-data assumptions. This plan includes quantitative evaluation using the E-value, assessment across alternative grace-period windows, comparisons across alternative model specifications, and comparisons across imputation approaches. Specifically, the potential threat posed by unmeasured confounding will be assessed through the calculation of the E-value (Gaster et al., 2023). As a sensitivity metric, the E-value defines the minimum strength of association an unmeasured confounder must share with both the exposure and the outcome to completely nullify the observed treatment effect (Haneuse et al., 2019). A higher E-value indicates that stronger unmeasured confounding would be required to explain away the observed association (Chung and Chung, 2023). Furthermore, this study incorporates sensitivity analyses of key design parameters within the TTE. The grace period refers to the allowed time window from “time zero” to actual treatment receipt; its purpose is to accommodate clinical practice while mitigating immortal time bias (Zhao et al., 2021). By comparing estimated effects of XYP on the primary outcomes under different grace-period settings, we will evaluate the sensitivity of the effect estimate to the time-window definition (Lumbard, 2025). Consistency of estimates across grace-period definitions would support the robustness of the findings. This multi-window assessment may also help contextualize results with respect to clinically plausible treatment-initiation times. To examine model dependence, the primary results will be compared with those from classical alternative approaches, including propensity score matching, propensity score stratification, and standard multivariable Cox or logistic regression, as appropriate (Bidulka et al., 2024; Cheng et al., 2025). Agreement of effect estimates across these models would reduce concern that conclusions depend on a particular modeling choice. Finally, sensitivity to missing-data assumptions will be assessed by comparing estimates from the primary machine-learning-based imputation with those from complete-case analysis and multiple imputation by chained equations using variable-type-appropriate models. Where feasible, departures from missing at random for key variables with substantial missingness will be explored using conservative controlled imputation or pattern-mixture specifications, supported by standard imputation diagnostics such as range checks and distributional comparisons (Bidulka et al., 2024).

Beyond estimating the average treatment effect, this protocol integrates causal forests and a staged dose-response analysis to characterize treatment effect heterogeneity and clinically relevant dosing patterns. While conventional research often emphasizes the average treatment effect, responses to pharmacologic interventions can vary substantially across individuals (Ling et al., 2023). To address this, we will use causal forests, a random-forest-based method for estimating heterogeneous treatment effects, to estimate the CATE across patient profiles (Feuerriegel et al., 2024). Using high-dimensional baseline clinical features, causal forests may help identify subgroups more likely to benefit from XYP. By providing an empirical description of patient heterogeneity, this approach may support hypothesis generation regarding which patients benefit most and inform subsequent confirmatory evaluation. In parallel, optimizing how XYP is administered is clinically important, particularly with respect to dosing, because efficacy and safety may both depend on exposure intensity. In routine practice, daily XYP doses vary widely, ranging from 50 mg to 500 mg (Li et al., 2023); and the dose-response relationship for severe pneumonia remains uncertain. To address this gap, we prespecify a multi-stage, nonlinear dose-response analysis. Rather than dichotomizing exposure, we will first use RCS in an exploratory step to describe potential nonlinear associations between continuous dose and outcomes. We will then define clinically interpretable dosing strategies, informed by RCS-derived change points and the real-world dose distribution, and evaluate their comparative effects within the target trial emulation (TTE) framework. This staged approach is intended to reflect real-world prescribing complexity while producing evidence that may be more directly relevant to clinical interpretation and future research.

Despite the rigor of TTE-based causal inference, several limitations should be acknowledged. First, EHR-derived RWD may pose data quality challenges because data are collected primarily for clinical and administrative purposes rather than research; missingness, measurement variability, heterogeneous coding practices, irregular timing of laboratory measurements, and misclassification can occur. These limitations are particularly relevant in drug intervention studies targeting specific high-risk populations, because frailty, functional status, polypharmacy, subtle changes in disease progression, clinician judgment, and contraindications to specific therapies may not be fully captured in structured EHR fields. Although we prespecify data curation procedures and apply machine learning-based imputation, residual bias due to data limitations may remain. Second, residual confounding and selection bias cannot be completely eliminated in this EHR-based observational study, despite the use of time-zero alignment, a new-user design, adjustment for a broad set of baseline covariates, and complementary causal inference methods. Residual confounding may affect causal-effect estimation in either direction. For example, if XYP is preferentially prescribed to patients with more severe or rapidly deteriorating disease, its treatment effect may be underestimated or biased toward harm; conversely, if clinicians avoid XYP in frail elderly patients or in patients with substantial baseline hepatic or renal dysfunction, its apparent effectiveness and safety may be overestimated. Therefore, the treatment-effect estimates should be interpreted under the prespecified identification assumptions and with caution. Third, machine learning-based heterogeneity findings, particularly subgroup profiles derived from causal forests, are exploratory and require confirmation in independent datasets before clinical implementation. Fourth, the use of a provincial database may limit external validity. Because clinical pathways, prescribing practices, and drug accessibility can vary across regions and health systems, extrapolation beyond the study setting should be made with caution.

This TTE protocol aims to systematically evaluate the real-world effectiveness and safety of XYP combined with standard care versus standard care alone in patients with severe pneumonia. The primary effect estimates will be interpreted under the prespecified causal identification assumptions and will focus on the primary outcomes, including 28-day all-cause mortality and in-hospital mortality. Causal forest analysis, exploratory subgroup analyses, and dose-response analysis will be interpreted as exploratory rather than confirmatory, and therefore should not be regarded as definitive evidence for treatment-effect heterogeneity or an optimal dosing regimen. Overall, this study is expected to provide real-world evidence to inform future evaluation of the role of XYP in the comprehensive management of severe pneumonia and to provide a replicable framework for generating real-world evidence for other TCM interventions.
