# Supplementary material for: Evaluating the effectiveness and safety of Xiyanping injection for severe pneumonia: a target trial emulation protocol using real-world data
Source: Front Pharmacol. 2026 Aug 6;17:1862861. doi: 10.3389/fphar.2026.1862861 (PMC13490165; doi:10.3389/fphar.2026.1862861)
Supplement: Supplementary file 1 [file DataSheet1.pdf]

ConPhyMP checklist of information for reporting plant material and its initial processing [1,2] (relevant for all studies on medicinal and food plants including extract types A, B, and C)

SECTION/TOPIC	ITEM NO.	CHECKLIST ITEM	YES	NO	NOT APPLICABLE	PAGE NO., IF ANY
Title and abstract	1	A clear and concise title including an informative abstract and balanced summary.	✓	○	○	and Abstract
Description of the botanical drug and taxonomic authentication	2	Botanical or morphological authentication of the plant material (desirable is a combination with DNA barcoding, e.g., PCR, RFLP, genome sequencing) and the information must be included in a separate section of Material and Methods, if applicable, combined with the information required under item 3:	✓	○	○	Sect. 2.4.2)
Description of the extract and extraction process	3	A separate section in Material and Methods, covers the relevant information on the material investigated, including the full species name(s), authorites and family; e.g. *Salvia miltorrhiza* Bunge [Lamiaceae; *Salviae miltorrhizae radix et rhizoma*], and on the processing and extraction of the crude drug including the traditional processing of the material used medicinally (fumigation, steaming, roasting, cooking, frying, etc.).	✓	○	○	Methods(Sect
Documentation of the legal basis for collection and processing	4	Full compliance with the Nagoya protocol, CITES, and all associated treaties including phytosanitary regulations.	✓	○	○	Sect. 2.4.2)
Description of product characteristics, in case of a finished (commercial) product	5	Information on the characteristics of the commercial products including batch number and date of production/best by information and regulatory status.	✓	○	○	Sect. 2.4.2)

Note: Please also include here the following information about your submitted manuscript:

Name of the journal:	Frontiers in Pharmacology
Date of the enquiry:	April 13, 2026
Title of the manuscript:	Target Trial Emulation Protocol Using Real-World Data
List of the authors:	, ZHANG Qiang, BAN Chengjun, XIE Yanming, WANG Zhifei

[1] Please acknowledge/cite this as follows: Heinrich M, Jalil B, Abdel-Tawab M, Echeverria J, Kulić Ž, McGaw LJ, et al. Best Practice in the chemical characterisation of extracts used in pharmacological and toxicological research—The ConPhyMP—Guidelines. Frontiers in Pharmacology. 2022;13:953205. https://doi.org/10.3389/fphar.2022.953205

[2] We strongly recommend reading this checklist in conjuncton with ConPhyMP 2022 explanaton and elaboraton for important clarifications on all items. If relevant, we also recommend after reading Heinrich et al. (2020) Best practice in research—Overcoming common challenges in phytopharmacological research. Journal of Ethnopharmacology. 2020;246:112230. https://doi.org/10.1016/j.jep.2019.112230
